# Evaluation of acute ocular toxicity after definitive-intent radiation therapy in canine sinonasal tumors

**DOI:** 10.1371/journal.pone.0329073

**Published:** 2025-08-11

**Authors:** Jonas Brückner, Carla Rohrer Bley, Antonella Rampazzo, Sergejs Unterkirhers, Simon Pot, Valeria Meier

**Affiliations:** 1 Clinic for Radiation Oncology & Medical Oncology, University Animal Hospital, Vetsuisse Faculty, University of Zurich, Zurich, Switzerland; 2 Ophthalmology Section, University Animal Hospital, Vetsuisse Faculty, University of Zurich, Zurich, Switzerland; 3 Department of Physics, University of Zurich, Zurich, Switzerland; 4 Institute for Radiotherapy, Hirslanden Clinic, Zurich, Switzerland; Colorado State University, UNITED STATES OF AMERICA

## Abstract

Acute toxicity and survival were documented in dogs undergoing radiotherapy for sinonasal tumors. However, few studies specifically investigated ocular toxicity, resulting in limited data on radiation tolerance thresholds. This study aimed to quantify acute ocular toxicity, compare toxicity levels using three different scoring systems, and establish practical preliminary dose-response limits. Dogs with sinonasal tumors treated with a definitive-intent 10-fraction radiotherapy protocol were included if they underwent prospective ophthalmic examinations, both prior to radiotherapy and at least once within three months following treatment. Ocular toxicity was assessed using three scoring systems: 1) Modified McDonald-Shadduck (evaluated by ophthalmologists), 2) Veterinary Radiation Therapy Oncology Group (VRTOG) 1.0 (evaluated by radiation oncologists), and 3) VRTOG2.0 (evaluated retrospectively). Radiation doses to each eye, retina, cornea, lacrimal, and accessory lacrimal gland were documented, and adherence to the new institutional ocular dose constraints was analyzed. Seventy client-owned dogs (140 eyes) were enrolled between 2016 and 2024, yielding 241 ophthalmic examinations. Clinically relevant ocular toxicity was identified in 28 eyes (20%) according to the modified McDonald-Shadduck system, 14 eyes (10%) using the VRTOG1.0 system (grade ≥2), and in 15 eyes (11%) using VRTOG2.0 toxicity (grade ≥3). Adherence to our institutional constraints resulted in low rates of clinically relevant toxicity: only 0.7% of eyes according to VRTOG 1.0 and 1.4% according to VRTOG2.0. TD5% and TD50% risk thresholds were established to facilitate optimized planning and reduced ocular risks. In conclusion, all dogs showed rather mild and only rarely clinically relevant ocular toxicity. Clinically relevant results of the very detailed modified McDonald-Shadduck scoring system seem to be accurately reflected by the VRTOG2.0 scoring system, which is more practical for daily clinical assessments. Detailed ocular evaluations are recommended primarily for dogs with clinically relevant VRTOG2.0 toxicity. Adherence to institutional ocular dose constraints significantly minimizes the risk of clinically relevant ocular toxicity.

## Introduction

Radiation therapy (RT) is the standard treatment for sinonasal tumors in dogs, offering effective local tumor control and improved survival outcomes [1–9]. However, the close anatomical relationship between sinonasal structures and ocular tissues presents a challenge, as radiation exposure can lead to acute and late-stage ocular toxicity. Acute toxicity occurs within the first three months following irradiation, predominantly affecting structures with high proliferative and regenerative capacities. In the periocular region, this includes the lacrimal glands, conjunctiva, and cornea [10–13]. Such acute effects commonly manifest as discomfort or can even cause pain but are generally reversible. In contrast, late toxicity is generally irreversible and may develop months to years after treatment and arises from chronic tear film deficiencies, degenerative changes, fibrosis or vascular damage in tissues with a low cellular turnover. These late ocular complications can ultimately lead to vision impairment, or the need for enucleation [14–16].

Although numerous studies have evaluated the efficacy of fractionated radiation therapy for sinonasal tumors in dogs, relatively few have specifically examined radiation-induced ocular toxicity [6,7,17]. Early studies, which used outdated, non-conformal radiation techniques, reported severe ocular damage, including globe perforation and permanent vision loss, as summarized by Wolf et al. [11]. Advanced radiation delivery methods, particularly fractionated intensity-modulated radiation therapy (IMRT), have substantially reduced both the prevalence and severity of ocular toxicity. Despite these improvements, comprehensive dose guidelines for canine ocular structures are still lacking [6,7,11,17]. Establishing ocular dose constraints would facilitate the development of safer and more effective treatment protocols, potentially enabling dose escalation and improving the still modest tumor control for canine sinonasal tumors while minimizing ocular toxicity.

In human radiation oncology, specific dose constraints for ocular structures are well established albeit fractionation and dose are different than in dogs [18,19]. In dogs, however, only few tolerance limits are available or derived from studies with small sample sizes: For instance, with a protocol of 10 × 4.2 Gy IMRT only mild, reversible ocular effects were reported when 60% of the eye received ≤ 15 Gy [7]. Another study using the same protocol found a significantly increased risk of keratoconjunctivitis sicca (KCS) at mean lacrimal gland dose above 33.1 Gy, whereas no dog developed KCS at doses below 20 Gy [20].

Veterinary radiation oncologists commonly assess treatment-related toxicity with the Veterinary Radiation Therapy Oncology Group (VRTOG) scoring system [15]. This system was recently updated to VRTOG2.0 to provide a more detailed and differentiated scoring of toxicity in organs at risk (OAR) [16]. However, because this system is typically completed by radiation oncologists themselves, ophthalmologic detail remains limited. A modified McDonald-Shadduck scoring system [21] has been used in prior studies for a more comprehensive ophthalmologic evaluation of ocular toxicity [17]. Integrating detailed ocular assessments into veterinary radiation oncology could improve our understanding of dose-related ocular toxicity and aid in the refinement of treatment protocols.

This prospective study evaluates acute ocular toxicity in dogs treated with a 10-fraction definitive-intent radiation therapy protocol. Ocular toxicity was prospectively assessed by veterinary ophthalmologists using the modified McDonald-Shadduck system at baseline and up to three months post-irradiation. Concurrent assessments were performed prospectively by veterinary radiation oncologists using the VRTOG1.0 grading system. Grading with the new VRTOG2.0 system was retrospectively added. Additionally, ocular organs at risk including the ocular globe, cornea, retina, lacrimal and accessory lacrimal glands were contoured in detail, and analyzed to establish dose-response relationships.

The primary aim was to quantify acute ocular toxicity following highly conformal, definitive-intent radiation therapy with a 10-fraction protocol, compare the three different scoring systems, and establish practical preliminary dose constraints to improve future radiation therapy planning for canine sinonasal tumors.

## Materials and methods

### Inclusion criteria and patient characteristics

Client-owned dogs were included into the study if they underwent radiation therapy as part of their regular treatment for a sinonasal tumor (any cytological/ histopathological entity or based on imaging characteristics) at the Clinic for Radiation Oncology & Medical Oncology, University Animal Hospital, University of Zurich, Switzerland between January 2016 and November 2024 and underwent complete ophthalmological examinations. Inclusion required a baseline ophthalmological examination prior to radiation therapy and at least one follow-up within three months, conducted as part of previous [1,17] or ongoing trials. Owner consent and ethical approval from the Animal Ethics Council of the Cantonal Veterinary Office of Zurich, Switzerland (ZH075/17 [1,17], ZH202/2022) were obtained before treatment. Radiation protocol adaptations used in the clinical trials mentioned above were carefully calculated beforehand, deemed safe for clinical implementation and the Animal Ethics Council therefore judged those trials to have a maximal severity degree of 1 (with available degrees from 0 to 3). No treatment-related deaths were expected and therefore no humane endpoints were defined. Information was collected regarding signalment, brachycephalic versus non-brachycephalic head conformation, tumor type and tumor stage according to the modified Adams staging system [9], and comorbidities.

### Treatment setup and planning

Treatment simulation, anesthesia, contouring, positioning and planning followed previous protocols [1,17]. All dogs received daily image-guided IMRT or volumetric modulated arc therapy using a 6 MV linear accelerator, with planning performed in the Eclipse Treatment Planning System (version 10.0, 15.1 or 17.0). Prior to December 2023, dogs were treated using a Clinac iX linear accelerator (Varian, Palo Alto, California, USA), afterwards using a TrueBeam linear accelerator (Varian, Palo Alto, California, USA). Treatments were delivered in 10 fractions over two consecutive weeks (Monday to Friday). Dose per fraction and special techniques (simultaneously integrated boost, heterogeneous planning) were documented. Treatment plans, created by a board-certified veterinary radiation oncologist (CRB, VM), were approved by a certified medical physicist, verified dosimetrically using a phantom (Octavius-PTW, Freiburg, Germany), and met institutional and federal quality assurance guidelines [22,23]. For the post-hoc dose comparisons, all treatment plans were recalculated using the same algorithm (AAA version 15.1) and grid size (0.1 cm).

### Ocular examinations, scoring and medical management

A board-certified veterinary ophthalmologist (SP, AR) conducted complete ocular examinations, including a baseline examination prior to radiation therapy, as previously described [17]. The examination protocol and scoring sheet are shown in S1 and S2 Fig, the material used in S1 Table. Follow-up examinations were recommended at the end of radiation therapy and 1, 2, 3, 8, and 12 weeks post-treatment for pilot study participants [17], and at 3 and 12 weeks for later cases. Pathologies in the anterior and posterior segment of the eye were graded using a modified McDonald-Shadduck scoring system [17]. All ocular abnormalities were documented and evaluated for possible relation to radiation therapy by a member of the radiation oncology team (JB) and a board-certified ophthalmologist (SP). Ocular abnormalities were deemed clearly unrelated to radiation therapy if: 1) present at baseline without worsening afterwards, or if 2) deemed to be of a congenital (and therefore permanent) nature. Such abnormalities were only scored as toxicity at one of the follow-up examinations if they increased (e.g., if a grade 1 ‘toxicity’ was present at baseline, it was counted as toxicity only if it increased to grade 2 or a higher grade). Clinically relevant toxicity was defined as modified McDonald-Shadduck grade 1 if there were only two scoring grades (grade 0: not present, grade 1: abnormality present, e.g., retinal hemorrhages). In cases with more scoring criteria with grade 0–2 (grade 0: none, grade 1: mild, grade 2: intense, e.g., conjunctival chemosis), grade 2 was defined as clinically relevant. If there were even more scoring criteria with grade 0–4, clinically relevant toxicity was defined as grade ≥3 (e.g., mucopurulent discharge, marked loss of corneal transparency, area of pigmentation >50%).

Starting from the first day of radiation therapy, 33 dogs (47%) treated between January 2016 and May 2020 received 0.2% cyclosporine (Optimmune, MSD Animal Health, Switzerland) and vitamin A eye ointment (Vitamin A Blache, Bausch&Lomb, Berlin, Germany), each administered twice daily into both eyes by the dog owner and recommended to be given at least 30 minutes apart. Vitamin A was given first in the morning and last in the evening and cyclosporin was given in-between. After June 2020, cyclosporine was replaced by artificial tears (Ocry-Gel, Virbac, Virbac (Switzerland) AG, Switzerland) twice daily for 37 dogs (53%). All ocular ointments were discontinued at the 3-week follow-up if the Schirmer tear test (STT) and Tear Film break-up time (TBUT) were normal. The respective eye medication was the standard of care at the clinic at this time.

### Radiation toxicity scoring

Starting on the last day of radiation therapy, acute ocular radiation effects of each dog were prospectively graded by a veterinarian of the radiation oncology team (JB) under a radiation oncologist’s supervision (CRB, VM) using the VRTOG1.0 criteria with grades 0–3 [15]. For the purpose of this study, acute ocular radiation effects were retrospectively graded from grade 0–5 using the VRTOG2.0 criteria by a veterinarian of the radiation oncology team (JB) under supervision of a board-certified radiation oncologist (VM) based on patient records (McDonald-Shadduck scores and reports in the clinic system) and images [16]. Clinically relevant toxicity was defined as VRTOG1.0 grade ≥2 (grade 2: KCS, moderate conjunctivitis necessitating therapy; grade 3: severe keratitis with corneal ulceration and/or loss of vision, glaucoma) and VRTOG2.0 grade ≥3 (grade 3: severe conjunctival hyperemia, nonsevere keratitis, neovascularization ≤50% of surface, decreased tear production necessitating tear stimulant therapy, blepharospasm; grade 4: severe keratitis with infiltrate or infection, corneal stromal loss with uveitis, neovascularization >50% of surface, STT = 0, nonresponsive to tear stimulant therapy, enucleation or surgery needed; grade 5: toxicity resulting in death or euthanasia).

### Contouring

**Fig 1:**
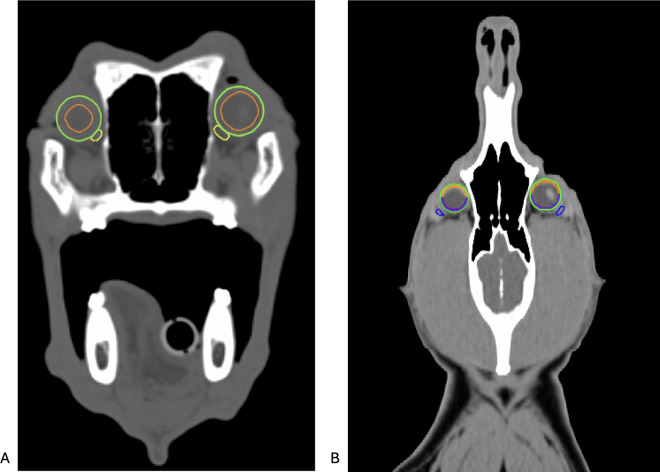
Contouring of the ocular organs at risk in the treatment planning system. Ocular structures were contoured in the treatment planning system to allow for detailed dose reporting: Eye (green), cornea (orange), retina (purple), accessory lacrimal gland (yellow), lacrimal gland (blue). A: transversal view, B: sagittal view.

In order to report the radiation dose received by each ocular structure and look for association with toxicity as described in the chapter below, all ocular structures needed to be contoured in the treatment planning system. If this had not already been done during the actual treatment planning process for each dog (e.g., accessory lacrimal glands were not routinely contoured) or if the contouring did not adhere to the contouring guidelines for this study as described below, ocular structures were newly contoured by a veterinarian of the radiation oncology team (JB) and approved by a board-certified veterinary radiation oncologist (VM). Contouring followed previous study guidelines with adaptations as follows [17,24] and as shown in Fig 1: The ocular globe was contoured as round structure with the 3D brush tool, with freehand adjustments for anatomic variations. The retinal and corneal structures were segmented in high-resolution, automatically contoured as 1 mm internal ring, with the cornea defined anteriorly and the retina posteriorly for ease. Lacrimal and accessory lacrimal glands were contoured on the co-registered post-contrast images. The lacrimal glands were contoured as flattened, contrast-enhancing structures located dorsotemporally of the eye. The accessory lacrimal glands were contoured as contrast-enhancing structures rostral and medial to the eye.

### Dose reporting

OAR volumes and radiation doses were reported for D2% (near maximum dose_,_ dose to 2% of the OAR volume) and D50% (mean dose). Adherence to institutional ocular constraints was assessed. Those constraints were developed for our most recent clinical trial where dose maxima up to 130% were allowed if OAR constraints were met. Constraints were based on previous veterinary data if available (eye [7], lacrimal glands [20]) or human data as summarized in Wolf et al. [11] and included: cornea maximum dose (Dmax) ≤35.4 Gy, retina Dmax ≤32.1Gy, lacrimal glands D50% ≤ 20 Gy, eye D60% ≤ 15 Gy for each organ at risk separately. The accessory lacrimal glands were not taken into account.

### Statistical analysis

Descriptive statistics were used to evaluate dog and tumor characteristics, eye examination parameters and dose-volume parameters. When appropriate, data were tested for normality using the Shapiro-Wilk normality test. Values were expressed as mean (± standard deviation (SD)) in case of normal distribution or as median and range in case of non‐normal distribution. Results of statistical tests with p < 0.05 were considered significant. All statistical analyses were performed using either GraphPad Prism version 10 for Windows (GraphPad Software, La Jolla CA, USA, www.graphpad.com) or Python (Pyton Software Foundation, version 3.12.1). The Python libraries utilized included pandas, NumPy, SciPy, statsmodels, scikit-learn, and Matplotlib. Differences in intraocular pressure (IOP), STT, and TFBUT values were evaluated using T-tests or Mann-Whitney tests (for comparisons between cyclosporine vs no cyclosporine treatment groups at the various recheck time points) and by fitting a mixed model (for multiple comparisons between time points). Differences in dose-volume parameters among the radiation therapy protocols were evaluated using the Kruskal–Wallis test. When significant differences were observed, pairwise comparisons were conducted with Bonferroni correction for multiple testing. Ocular toxicity outcomes were summarized using frequency counts and proportions according to each scoring system (McDonald–Shadduck, VRTOG1.0, VRTOG2.0). To evaluate the association between increasing radiation dose and risk of ocular toxicity, univariable logistic regression models were constructed, providing odds ratios (OR) and 95% confidence intervals (CI) per 1 Gy increment for specific dose-volume parameters (e.g., eye D2%, cornea D50%). An OR >1 indicated a higher risk of clinically relevant toxicity (e.g., VRTOG1.0 grade ≥2, VRTOG2.0 grade ≥3) per additional 1 Gy increment. Exploratory changepoint analyses and piecewise (segmented) logistic regression were employed to identify potential dose thresholds at which ocular toxicity risk significantly increased. For each ocular structure assessed, potential cutoff points were systematically evaluated to detect a “knot” representing a significant change in the slope of the dose-toxicity relationship. The optimal cutoff point was determined based on model fit criteria, including deviance and likelihood ratio tests, and by maximizing the difference in toxicity incidence between groups below and above the identified dose threshold. This methodology was applied separately for key dose-volume metrics such as eye D50%, retina D50%, and cornea D50% to ascertain whether exceeding specific dose thresholds corresponded with a marked increase in toxicity.

## Results

### Patient and treatment protocol characteristics

Seventy dogs with 140 eyes were included into the study between January 2016 and November 2024. Information on dog and tumor characteristics and radiation therapy protocols used is presented in Table 1. All enrolled dogs underwent 10-fraction definitive-intent radiation therapy protocols, including 1) a standard 10 x 4.2 Gy protocol with homogeneous dose distribution (n = 32, participation in ongoing or previous studies [1]), 2) a protocol with 10 x 4.2 Gy to the planning target volume plus an additional simultaneously integrated boost (SIB) of +20% to the gross tumor volume (n = 15, participation in previous studies [1,17]) or 3) a protocol with 10 x 4.2 Gy allowing heterogeneous dose distribution (dose maxima up to 130% of the prescribed dose) provided OAR constraints were met (n = 23, ongoing study). No concurrent or adjuvant chemotherapy was administered. Several dogs had secondary neoplastic diseases or age-related comorbidities (osteoarthrosis, cardiac disease, partial laryngeal paralysis), but none had hormonal disorders predisposing them to ocular disease. Brachycephalic breeds accounted for 11% of enrolled dogs.

**Table 1:** Patient characteristics.

Characteristic	Number of dogs (%)
**Age**	
median (range) [years]	9.5 (3.7-15.8)
**Weight**	
median (range) [kg]	24.2 (3.3-60.0)
**Sex**	
Female	7 (10)
Female spayed	24 (34)
Male	11 (16)
Male castrated	28 (40)
**Breed**	
Non-brachycephalic	62 (89)
Brachycephalic	8 (11)
**Type of tumor**	
Carcinoma	39 (56)
Sarcoma	21 (30)
Esthesioneuroblastoma	1 (1)
Benign	
Extensive polyps	2 (3)
Angiofibroma	1 (1)
Unknown	6 (9)
**Stage** (modified Adams staging system [9])	
I	14 (20)
II	16 (23)
III	18 (26)
IV	22 (31)
**Primary tumor site**	
Right nasal cavity	35 (50)
Left nasal cavity	22 (31)
Bilateral	10 (14)
Originating primarily from frontal sinus	3 (4)
**Protocol**	
10x4.2Gy standard protocol	32 (46)
10x4.2Gy with simultaneously integrated boost	15 (21)
10x4.2Gy with heterogeneous dose distribution	23 (33)
**Eye medication**	
0.2% cyclosporine (Optimmune) & vitamin A eye ointment	33 (47)
Artificial tears (Ocry-Gel) & vitamin A eye ointment	37 (53)

### Ocular examinations

Two hundred and forty-one ocular examinations were performed in total. Baseline ocular examinations were available for all dogs as defined by the inclusion criteria. In 35.7% (25/70) of dogs, ocular abnormalities were seen at baseline, which remained the same or decreased at the follow-up examinations and were therefore classified as unrelated to radiation therapy. Follow-up ocular examinations and concurrent VRTOG1.0 toxicity scoring were available on the day of the last RT fraction in 76.1% of dogs, at 1 week after RT in 14.3%, at 2 weeks in 11.4%, at 3 weeks in 82.9%, at 2 months in 12.9%, and at 3 months in 55.7% of dogs.

Results of IOP, STT, and TBUT at various time points are shown in the S3 Fig. Results of ocular McDonald-Shadduck scoring over time are shown in Table 2. Intraocular pressure values were significantly lower at the end of RT recheck examination, compared to the baseline, three-week and three-month examinations (p < 0.0001). Schirmer tear test values were significantly lower at the three-month recheck examination, compared to the baseline, end of RT and three-week examinations (p < 0.0001). No significant differences in TFBUT measurements were observed between any of the examination time points with >50% attendance (p values between 0.07 and 1). No significant differences were observed at any of the recheck examination time points in either STT and TBUT measurements between the patient cohorts that received twice daily topical 0.2% cyclosporine (Optimmune, MSD Animal Health, Switzerland) ointment and vitamin A ointment (Vitamin A Blache, Bausch&Lomb, Berlin, Germany) and patients that received artificial tears (Ocry-Gel, Virbac, Virbac (Switzerland) AG, Switzerland) and vitamin A ointment during and after their radiation therapy (data not shown, p values for the comparisons at the various time points ranging between 0.1 and 1). Complete ocular examination results, toxicity score and radiation dose data are deposited in an open repository (Harvard Dataverse, https://www.editorialmanager.com/pone/l.asp?i=75371566&l=PWWHPXKF).

**Table 2:** McDonald-Shadduck scoring of all dogs over time.

	Baseline	End of radiation therapy	3 weeks	3 months
**Intraocular pressure**	13 (8-26)	10.6 (±2.8)	11.7 (±2.8)	13.5 (±3.4)
median (range)^a^ [mmHg]				
normal range: 10–25 mmHg				
**Schirmer tear test**	21 (12-36)	22 (10-35)	22 (13-35)	19 (2-28)
median (range) [millimeter/minute]				
normal range: 10–25 mm/min				
**Tear film breakup time**	15.8 (±3.7)	16 (8-20)	12.8 (±3.8)	14.3 (±4.1)
median (range)^a^ [seconds]				
normal range: > 10 sec				
**Corneal pigmentation**				
Grade 0	130 (93%)	76 (81%)	89 (77%)	61 (82%)
Grade 1	10 (7%)	17 (18%)	25 (22%)	11 (15%)
Grade 2	0	0	1 (1%)	1 (1%)
Grade 3^b^	0	1 (1%)	1 (1%)	1 (1%)
Grade 4^b^	0	0	0	0
**Conjunctival chemosis**				
Grade 0	133 (95%)	89 (95%)	105 (91%)	72 (97%)
Grade 1	5 (4%)	4 (4%)	10 (9%)	2 (3%)
Grade 2^b^	1 (1%)	1 (1%)	1 (1%)	0
**Conjunctival congestion**				
Grade 0	131 (94%)	86 (91%)	90 (78%)	64 (86%)
Grade 1	7 (5%)	7 (7%)	23 (20%)	10 (14%)
Grade 2^b^	1 (1%)	1 (1%)	3 (1%)	0
**Conjunctival discharge**				
Grade 0	126 (90%)	85 (90%)	92 (79%)	64 (86%)
Grade 1	11 (8%)	8 (9%)	11 (9%)	6 (8%)
Grade 2	1 (1%)	0	9 (8%)	4 (5%)
Grade 3^b^	0	0	6 (5%)	0
Grade 4^b^	1 (1%)	1 (1%)	0	0
**Corneal opacity**				
Grade 0	124 (88%)	76 (81%)	86 (75%)	56 (76%)
Grade 1	15 (11%)	17 (18%)	27 (24%)	15 (20%)
Grade 2	1 (1%)	1 (1%)	1 (1%)	1 (1%)
Grade 3^b^	0	0	0	0
Grade 4^b^	0	0	0	2 (3%)
**Area of corneal opacity**				
Grade 0	128 (91%)	76 (81%)	86 (75%)	58 (78%)
Grade 1	11 (8%)	17 (18%)	26 (23%)	13 (18%)
Grade 2	0	0	2 (2%)	1 (1%)
Grade 3^b^	0	1 (1%)	0	0
Grade 4^b^	1 (1%)	0	0	2 (3%)
**Corneal neovascularization**				
Grade 0	138 (99%)	92 (98%)	110 (95%)	67 (92%)
Grade 1	2 (1%)	2 (2%)	6 (5%)	5 (7%)
Grade 2^b^	0	0	0	1 (1%)
**Anterior chamber/aqueous flare**				
Grade 0	140 (100%)	94 (100%)	113 (99%)	71 (100%)
Trace	0	0	0	0
Grade 1	0	0	1 (1%)	0
Grade 2–3	0	0	0	0
Grade 4	0	0	0	0
**Aqueous cell**				
Grade 0	138 (99%)	94 (100%)	109 (96%)	71 (100%)
Trace	2 (1%)	0	5 (4%)	0
Grade 1	0	0	0	0
Grade 2–3	0	0	0	0
Grade 4	0	0	0	0
**Iris**				
Grade 0	120 (86%)	83 (88%)	100 (88%)	66 (96%)
Grade 1	20 (14%)	11 (12%)	12 (11%)	3 (4%)
Grade 2	0	0	1 (1%)	0
Grade 3	0	0	0	0
**Lens**				
Grade 0	70 (50%)	52 (55%)	54 (48%)	30 (42%)
Grade 1	70 (50%)	42 (45%)	59 (52%)	41 (58%)
**Vitreal cell**				
Grade 0	130 (94%)	83 (89%)	105 (94%)	68 (94%)
Trace	5 (4%)	5 (5%)	0	1 (1%)
Grade 1	2 (1%)	5 (5%)	5 (4%)	3 (4%)
Grade 2–3	0	0	0	0
Grade 4	2 (1%)	0	2 (2%)	0
**Vitreal degeneration**				
Grade 0	115 (83%)	84 (90%)	96 (86%)	60 (86%)
Grade 1	20 (14%)	7 (8%)	11 (10%)	7 (10%)
Grade 2	4 (3%)	2 (2%)	4 (4%)	3 (4%)
**Vitreal hemorrhage**				
Grade 0	137 (100%)	92 (100%)	111 (100%)	69 (99%)
Grade 1	0	0	0	1 (1%)
Grade 2	0	0	0	0
**Retinal hemorrhages**				
Grade 0	137 (98%)	86 (96%)	109 (98%)	62 (91%)
Grade 1^b^	2 (1%)	4 (4%)	2 (2%)	6 (9%)
**Retinal detachment**				
Grade 0	135 (99%)	91 (100%)	109 (98%)	70 (100%)
Grade 1	2 (1%)	0	2 (2%)	0
Grade 2	0	0	0	0
Grade 3^b^	0	0	0	0
Grade 4^b^	0	0	0	0
**Retinal tears/holes**				
Grade 0	138 (100%)	91 (100%)	111 (100%)	70 (100%)
Grade 1^b^	0	0	0	0
**Chorioretinitis lesion**				
Grade 0	136 (99%)	90 (99%)	110 (99%)	67 (96%)
Grade 1^b^	2 (1%)	1 (1%)	1 (1%)	3 (4%)
**Fluorescein**				
Grade 0	136 (97%)	93 (99%)	109 (94%)	69 (91%)
Grade 1^b^	4 (3%)	1 (1%)	7 (6%)	7 (9%)

^a^For data with normal distribution, mean ± standard deviation was reported.

^b^McDonald-Shadduck grade defined as clinically relevant.

### Ophthalmological and radiation toxicity scoring

**Fig 2:**
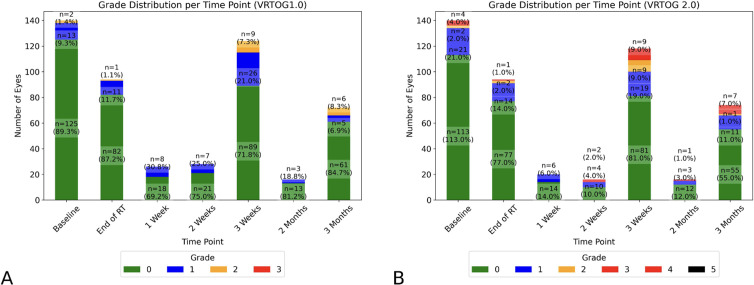
Acute radiation toxicity according to VRTOG1.0 and 2.0. Barplots visualizing the percentage of eyes (y-axis) with acute ocular radiation toxicity at the different time points (x-axis) for the A) VRTOG1.0 and B) VRTOG2.0 scoring system. The different grades (grade 0-3 for VRTOG1.0 and grade 0-5 for VRTOG2.0) are depicted with different colors. Clinically relevant toxicity (grade 2 VRTOG1.0, grade 3 VRTOG2.0) is depicted in yellow. No grade 3 VRTOG1.0 or grade 4 or 5 VRTOG2.0 toxicity was detected.

**Fig 3:**
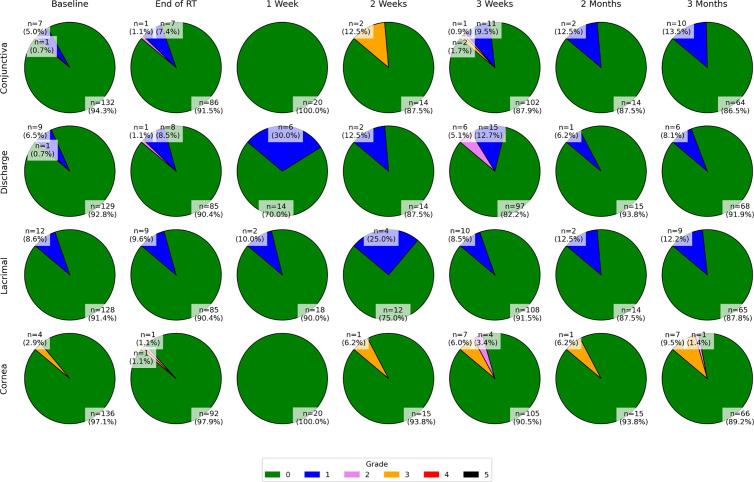
Acute VRTOG2.0 toxicity of the different ocular compartments over time. Acute VRTOG2.0 toxicity grades of the different ocular compartments (conjunctiva, ocular discharge, lacrimal gland, cornea) at the different follow-up examinations. Some dogs had ocular abnormalities at the baseline examination, which is shown in the first column. The different grades are depicted in color. Clinically relevant (grade 3) toxicity is depicted in yellow. No grade 4 or 5 toxicity was detected.

In terms of overall toxicity, 79 (56%) eyes showed an elevated grade above baseline at one of the follow-up examinations in any of the ocular structures according to the modified McDonald-Shadduck, 44 (31%) according to the VRTOG1.0 and 49 (35%) according to the VRTOG2.0 scoring system. Clinically relevant VRTOG1.0 toxicity (grade ≥2) at one of the follow-up examinations was detected in 14 eyes (10%), while clinically relevant VRTOG2.0 toxicity (grade ≥3) was observed in 15 eyes (11%) as shown in Table 3. Clinically relevant McDonald-Shadduck toxicity was observed in 28 eyes (20%) as shown in Table 2. Clinically relevant VRTOG1.0 grade ≥2 toxicity was detected in 1 eye (0.7%) and clinically relevant VRTOG2.0 grade ≥3 toxicity was detected in 2 eyes (1.4%) when our new dose constraints (described under Dose Reporting in the Material and Methods section) for that specific organ were met. The institutional constraints for the cornea were met in 69 eyes (49.2%), the constraints for the lacrimal glands in 116 eyes (82.9%), the constraints for the retina in 67 eyes (47.9%), and the constraints for the ocular globe were met in 79 eyes (56.4%). The VRTOG1.0 and VRTOG2.0 grade distribution at the different examination time points are visualized in Fig 2. A relatively small number of dogs was examined at 1 week, 2 weeks and 2 months after RT as visualized by the shorter barplots, and only 3 of the dogs were examined at all the time points. In total, 15 dogs had 2 ocular and clinical examinations, 28 dogs had 3 examinations, 17 dogs had 4 examinations, 4 dogs had 5 examinations, 3 dogs had 6 examinations, and 3 dogs had 7 examinations. The separated VRTOG2.0 grades for conjunctiva, ocular discharge, lacrimal gland and cornea are shown in the pie charts in Fig 3.

**Table 3:** Time points and outcome of clinically relevant VRTOG1.0 grade 2 and/ or VRTOG2.0 grade 3 toxicity.

	Baseline ocular abnormality present	Toxicity after radiation therapy	Ocular structure/ abnormality	Time point	Therapy	Outcome
Dog 1	Conjunctival congestion	Unilateral VRTOG1.0 grade 2, VRTOG2.0 grade 2	Unilateral ocular discharge	3 weeks	Lubricant & cyclosporin were continued	Resolved at next appointment
Dog 2, 3^a^, 4 and 15	No	Unilateral VRTOG1.0 grade 1, VRTOG2.0 grade 3	Unilateral fluorescein positivity	2 weeks (dog 2) 3 weeks (dog 15) 2 months (dog 3) 3 months (dog 4)	Lubricant & cyclosporin were continued	Improved at next appointment (no longer clinically relevant) under therapy
Dog 5	No	Unilateral VRTOG1.0 grade 2, VRTOG2.0 grade 1	Unilateral ocular discharge	3 weeks	Lubricant & cyclosporin were continued	Resolved at next appointment examination, cyclosporin was continued
Dog 6, 8 and 9	No	Unilateral (Dog 6 bilateral) VRTOG1.0 grade 2, VRTOG2.0 grade 0	Unilateral retinopathy (minor)	3 months	No change in therapy	Dog 6: stable Dog 7 & 9: improved at next appointment (no longer clinically relevant)
Dog 7 and 16	Dog 7: purulent discharge and conjunctival chemosis Dog 16: no changes	Bilateral VRTOG1.0 grade 2 and bilateral VRTOG2.0 grade 3	Bilateral reduced tear production (keratoconjunctivitis sicca)^b^	3 months (Dog 7) 3 weeks (Dog 16)	Dog 7: Immunosuppressive medical treatment, antibiotic eye medication Dog 16: Cyclosporin was continued	Dog 7: stable at next appointment, but exophthalmos due to progressive disease of nasal tumor Dog 16: improved at next appointment (no longer clinically relevant) under cyclosporin therapy
Dog 10^c^	Unilateral conjunctival congestion and discharge, bilateral corneal opacity and fluorescein positivity	Bilateral VRTOG1.0 grade 2, VRTOG2.0 grade 3 (suspected to be most likely immune-mediated or paraneoplastic in origin)	Bilateral conjunctival congestion Bilateral severe hyperemia of conjunctiva, uveitis	2 weeks 3 weeks	Immunosuppressive medical treatment, lubricant was continued	Improved at next appointment (no longer clinically relevant)
Dog 11	No	Bilateral VRTOG1.0 grade 1, VRTOG2.0 grade 3	Bilateral reduced tear production (keratoconjunctivitis sicca)^b^	3 weeks and 3 months	Cyclosporin was continued	Improved at next appointment (no longer clinically relevant) under cyclosporin therapy
Dog 12	No	Bilateral VRTOG1.0 grade 2, VRTOG2.0 grade 2	Bilateral purulent ocular discharge and blepharitis	3 weeks	Vitamin A eye ointment and cyclosporin were stopped, start with antibiotic eye medication & cleaning wipes for the eyelids	Improved at next appointment (only mild ocular discharge)
Dog 13	No	Unilateral VRTOG1.0 grade 0, VRTOG2.0 grade 3	Unilateral corneal fluorescein positivity	End of RT	No change in therapy (lubricant was given as planned until 3-week-re-check)	Improved at next appointment (no longer clinically relevant)
Dog 17	Conjunctival chemosis, congestion, discharge	Unilateral VRTOG1.0 grade 2, VRTOG2.0 grade 2	Unilateral ocular discharge	End of RT	No change in therapy (lubricant was given as planned until 3-week-re-check)	Improved at next appointment (no longer clinically relevant)
Dog 14 and 18	No	Bilateral VRTOG1.0 grade 0, VRTOG2.0 grade 3	Bilateral corneal fluorescein positivity	3 weeks (Dog 18) 3months (Dog 14)	Lubricant was continued	Stable at next appointment

^a^Brachycephalic dog.

^b^Mean dose (D50%) to both lacrimal glands in dogs with bilateral reduced tear production was as follows: dog 7 (24.4 Gy and 42.2 Gy), dog 11 (14.9 Gy and 12.0 Gy), dog 16 (17.7 Gy and 18.9 Gy).

^c^This dog had bilateral retinal detachment (grade 1 according to the modified McDonald-Shadduck scoring system) at baseline and at subsequent visits (most likely immune-mediated or paraneoplastic in origin), which was not counted as acute ocular toxicity as it was present at baseline and did not worsen during the period of the study and because retinal changes are only mentioned as late toxicity according to VRTOG1.0 and VRTOG2.0. The retina reattached at a re-check appointment following the time period of this study.

Dogs that developed clinically relevant VRTOG1.0 grade 2 and/ or VRTOG2.0 grade 3 toxicity are described in detail in Table 3. No dog developed severe acute VRTOG1.0 grade 3 or VRTOG2.0 grade ≥4 toxicity.

Clinically relevant corneal toxicity according to the McDonald-Shadduck scoring system was observed in 12.5% of eyes of brachycephalic dogs (n = 2/16) versus 0.8% of eyes of non-brachycephalic dogs (n = 1/124; p = 0.034). None of the other toxicity endpoints showed a significant association with brachycephalic status. There was no significant difference in clinically relevant outcomes (McDonald-Shadduck clinically relevant corneal, lacrimal, retinal, conjunctival toxicity, VRTOG1.0 eye toxicity grade ≥2, VRTOG2.0 any toxicity, VRTOG2.0 eye toxicity grade ≥3, VRTOG2.0 highest lacrimal toxicity) between the different RT treatment protocols.

### Radiation dose reporting

**Fig 4:**
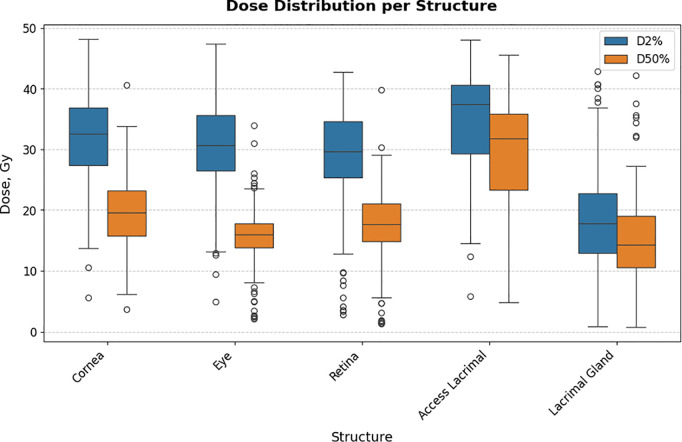
Boxplots depicting the dose distribution across all ocular structures. Boxplot depicting the dose distribution (near-maximum dose (D2%) depicted in blue, mean dose (D50%) depicted in orange) of the ocular structures cornea, eye, retina, accessory lacrimal (Access Lacrimal) and lacrimal gland.

**Fig 5:**
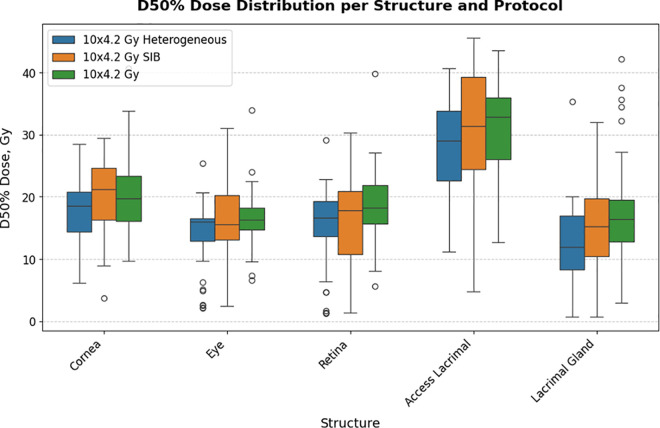
Boxplot of mean dose distribution per ocular structure and protocol. Boxplots depicting the D50% (mean) dose distribution for the cornea, eye, retina, accessory lacrimal (Access Lacrimal) and lacrimal gland. The different protocols are shown in different colors.

Boxplots illustrating mean (D50%) and near-maximum (D2%) radiation doses for each ocular structure and the mean dose per structure and protocol are shown in Figs 4 and 5, respectively. The accessory lacrimal glands were newly contoured for this study and no constraint was used to reduce dose during the former (actual) treatment planning process. This explains the higher accessory lacrimal gland dose in Figs 4 and 5.

A statistically significant difference in mean dose (D50%) to the cornea and lacrimal gland was noted among the three protocols according to the Kruskal–Wallis test (p = 0.005 and p = 0.036, respectively). Subsequent Bonferroni-corrected post hoc analysis showed that the difference was between the standard 10x4.2 Gy protocol and the protocol with heterogeneous dose distribution (17.5 + /-5.33) versus the protocol with SIB (20.45 + /-6.03) (p = 0.042) for cornea D50%, and between the standard 10x4.2 Gy protocol (17.11 + /-7.45) versus the protocol with heterogeneous dose distribution (12.34 + /-6.67) for D50% lacrimal gland (p = 0.003).

### Dose-toxicity associations and risk thresholds

**Fig 6:**
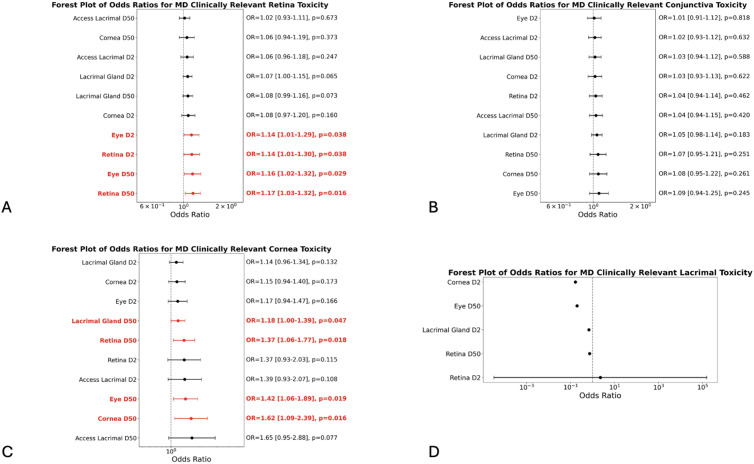
Forest plots of odds ratios for clinically relevant McDonald-Shadduck toxicity. Forest plots of odds ratios for clinically relevant McDonald-Shadduck toxicity for A) retina, B) conjunctiva, C) cornea, and D) lacrimal gland.

**Fig 7:**
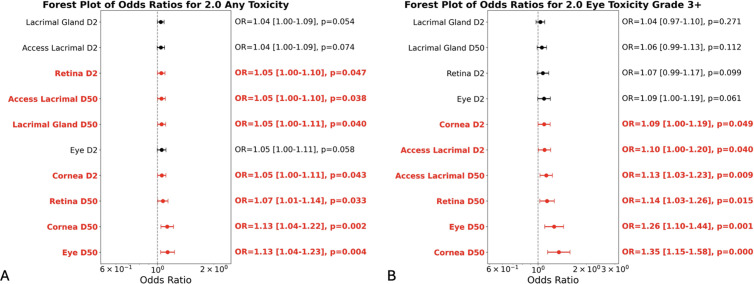
Forest plot of odds ratios for VRTOG2.0. Forest plots of odds ratios for VRTOG2.0 A) any toxicity and B) clinically relevant toxicity.

Odds ratios (OR) for the occurrence of any toxicity and for clinically relevant toxicity (McDonald-Shadduck, VRTOG1.0 and VRTOG2.0) were calculated based on the radiation doses delivered to different eye compartments as shown in Figs 6 and 7. For instance, the ORs for clinically relevant retinal toxicity (as scored with the McDonald-Shadduck grading system) associated with near-maximum (D2%) and mean (D50%) radiation dose to the whole eye and retina, were 1.14 (95% CI: 1.01–1.29), 1.16 (95% CI: 1.02–1.32), 1.14 (1.01–1.30), and 1.17 (1.03–1.32), respectively (Fig 6A). This indicates that each 1 Gy increase in dose (eye D2%, eye D50%, retina D2%, and retina D50%) is associated with a 14%, 16%, 14%, and 17% increase in the odds of toxicity to the retina, respectively. The ORs for clinically relevant toxicity (as scored with the VRTOG2.0 grading system) associated with mean radiation dose to the whole eye (eye D50%) and cornea (cornea D50%) were 1.26 (95% CI: 1.10–1.44) and 1.35 (95% CI: 1.15–1.58), respectively, corresponding to a 26% and 35% increase in the odds of clinically relevant toxicity for each 1 Gy increase in radiation dose (Fig 7b).

Table 4 summarizes the 5% risk thresholds (TD5%) for clinically relevant toxicity, while Table 5 lists 50% risk thresholds (TD50%). The TD5% represents radiation dose levels with a low risk (5% risk) of clinically relevant toxicity, while the TD50% threshold describes radiation dose levels associated with a markedly higher likelihood (50% risk). Already during the treatment planning process these TD5% and TD50% serve as critical reference points (rather than strict dose constraints) to guide dose distribution towards a clinically acceptable result for ocular organs at risk. The toxicity risk can therefore be foreseen for an individual dog, and the radiation therapy planned and steered into a direction with a lower risk, if needed.

**Table 4:** 5% Toxicity risk thresholds for clinically relevant ocular toxicity according to the McDonald-Shadduck and VRTOG2.0 toxicity scoring system.

Scoring system	Ocular structure with predicted clinically relevant toxicity	Dose-volume parameter	5% risk threshold dose (95%CI) [Gy]	P-value
VRTOG2.0	Eye	Retina D50%	18.3 (14.8 - 21.8)	0.001
	Eye	Eye D50%	12.8 (10.0–17.5)	0.001
	Eye	Cornea D50%	20.7 (17.0–36.6)	0.002
	Eye	Accessory Lacrimal Gland D50%	25.1 (21.3–51.6)	0.032
McDonald-Shadduck	Retina	Eye D50%	12.1 (9.4–16.2)	0.016
	Retina	Retina D50%	24.4 (14.7–34.0)	0.017
	Cornea	Eye D50%	22.3 (16.9–27.5)	0.019
	Cornea	Cornea D50%	30.5 (25.0–37.3)	0.039
	Retina	Retina D2%	15.3 (9.5–20.8)	0.028
	Retina	Eye D2%	30.1 (23.4–36.6)	0.036
	Retina	Eye D2%	17.7 (12.3–23.1)	0.015

**Table 5:** 50% Toxicity risk thresholds for VRTOG2.0 scoring system.

Scoring System	Type of Toxicity	Dose-volume Parameter	50% Risk Threshold Dose (95%CI) [Gy]	p-Value
VRTOG2.0	Any toxicity	Eye D50%	29.9 (25.4–34.4)	0.001
	Any toxicity	Cornea D50%	26.7 (21.3–32.1)	0.001
	Any toxicity	Retina D50%	25.6 (19.4–31.8)	0.003
	Clinically relevant eye toxicity	Eye D50%	25.7 (20.4–31.0)	0.005
	Clinically relevant eye toxicity	Cornea D50%	26.7 (17.4–36.2)	0.031

## Discussion

This study assessed acute ocular toxicity in dogs undergoing definitive-intent radiation for sinonasal tumors using ophthalmological exams and standard toxicity assessments. A dose-toxicity analysis correlated radiation doses with observed effects, as scored by the modified McDonald-Shadduck, VRTOG1.0 and VRTOG2.0 system. Adherence to institutional dose constraints was linked to a very low incidence of clinically relevant toxicity.

Increases in the toxicity scores above baseline were quite common and observed in 56% (McDonald-Shadduck), 31% (VRTOG1.0) and 35% (VRTOG2.0) of eyes. However, clinically relevant toxicity was less frequent: 20% using the detailed ophthalmological McDonald-Shadduck scoring system, and only 10% of VRTOG1.0 and 10.7% of VRTOG2.0 scores. This was even lower when our previously established dose constraints were met with 0.7% according to VRTOG1.0 and 1.4% according to VRTOG2.0.

The definition of clinically relevant toxicity was a clinical decision by the radiation oncology team, depending on how many grades were available and based on the subjective clinical impact. For the VRTOG1.0 scoring system with grade 0–3, we defined clinically relevant toxicity as grade ≥2 [15]. For the VRTOG2.0 scoring system with grade 0–5 we defined clinically relevant toxicity as grade ≥3. Grade 3 was described as *“medically significant changes that moderately impact daily activities”* by the VRTOG [16], even though this might only cause minor impact in daily activities in case of the eye (grade 3: severe conjunctival hyperemia, nonsevere keratitis, decreased tear production that can be treated with tear stimulant therapy). For the McDonald-Shadduck scoring system, definition of clinically relevant abnormalities was more difficult, as there were many different scoring criteria for each ocular structure (conjunctival congestion, chemosis, discharge; corneal opacity & area of corneal opacity, pigmentation, neovascularization; retinal detachment, tears, hemorrhages) and there was even a different number of grades available for each of those scoring criteria (e.g., grade 0–1 retinal hemorrhage; grade 0–2 conjunctival congestion; grade 0–4 conjunctival discharge). The authors are fully aware that the definition of clinically relevant toxicity, especially for the McDonald-Shadduck scoring system, was in part subjective and arbitrary, but it was a necessary tool for ease of statistical evaluation. In retrospect, we should have made an exemption of our rules (if scoring from grade 0–4 is available, clinically relevant toxicity was defined as grade ≥3) for retinal detachment, as any degree of retinal detachment is a serious event. Fortunately, however, this issue had no impact on our study, because no dog enrolled in the study experienced the occurrence of a retinal detachment during or following radiation therapy. For the remaining scores, it is possible that our definition was rather conservative and that the cut-off values for true clinically relevant toxicities could be set higher. However, the authors find it best to be careful as a good quality of life is of utmost importance in veterinary cancer patients.

The VRTOG2.0 scoring system [16] differentiates grade 0–5, but also uses attribution categories (unrelated, unlikely, possible, probable, definite) as a new feature compared to VRTOG1.0 [15]. For our study and for ease of statistical evaluation, we attributed all ocular changes (if higher than the baseline score and if not congenital) as *“definite”* and therefore *“clearly radiation therapy related”*. Even though, one dog (dog 10) would most likely have the attribution *“unrelated”* or *“unlikely related to radiation therapy”* as this dog was suspected to suffer from an immune-mediated disease or paraneoplastic syndrome. Assigning the attribution *“definite”*, which indicates a clear relation to radiation therapy might be overly conservative as some dogs had ocular abnormalities present prior to the start of radiation therapy, many dogs were elderly, and some might have developed decreased tear production due to repeated anesthetic events.

The highest toxicity grades identified were grade 2 according to the VRTOG1.0 grading system and grade 3 according to the VRTOG2.0 grading system. No VRTOG1.0 grade 3 or VRTOG2.0 grade 4 or 5 toxicity was documented. While no such severe toxicities were identified and no enucleation was necessary, some ocular abnormalities with the potential of being permanent were detected. Specifically, three dogs (4.3%) developed bilateral keratoconjunctivitis sicca VRTOG2.0 grade 3 (decreased tear production or qualitative tear abnormalities treated with tear stimulant therapy). This is treatable but often requires life-long medical management. Certain clinical signs observed at the three-month recheck examination might be associated with the observed decreased STT values at this time point. These signs included conjunctival chemosis (grade 1; 3% of patients), conjunctival congestion (grade 1; 14% of patients), conjunctival discharge (grades 1 and 2; 8 and 5% of patients), corneal neovascularization (grades 1 and 2; 7 and 1% of patients), and corneal pigmentation (grades 1, 2 and 3; 15, 1 and 1% of patients) (Table 2). Note that only grade 2 corneal neovascularization and grade 3 corneal pigmentation were defined as clinically relevant toxicity under the modified McDonald-Shadduck grading system, affecting only single patients at the three-month recheck examination (Table 2). In our cohort, all dogs with KCS demonstrated improved Schirmer tear test and/or improved qualitative tear film abnormalities under treatment with cyclosporin eye ointment and therefore a decreased toxicity grade at a subsequent visit, consistent with a therapy-responsive, well-controlled KCS (Table 3). Furthermore, we did not have VRTOG2.0 grade 4 toxicities of lacrimal glands (Schirmer tear test 0, non-responsive to tear stimulant therapy). Further analysis of the D50% dose of the lacrimal glands of dogs affected with KCS in our study revealed that two of the dogs received a D50% > 20 Gy and one dog < 20 Gy. Poirier et al. also used a 10-fraction protocol and recommended a D50% lacrimal dose <20 Gy (both, the lacrimal glands and the accessory lacrimal glands were contoured) as the D50% dose for developing KCS was 23.8 Gy and no dogs that received a D50% dose <20 Gy developed KCS, versus 71% with >20 Gy [20]. As the accessory lacrimal gland contributes to approximately 30–40% of tear production, it seems important to not only consider the lacrimal glands, but the accessory lacrimal glands as well [25,26]. A significant increase in corneal toxicity according to the McDonald-Shadduck scoring system was found in brachycephalic dogs. This apparent difference – and the absence of any differences in the other toxicity measures – should be interpreted with caution, as only 16 of the 140 eyes in our series were from brachycephalic dogs, limiting the power to detect true effects. Most of the lens changes observed at the baseline and later examinations in +/- 50% of eyes were senile or age-related cataracts, with a previously described prevalence of 50% in dogs with a mean age of 9.4 years [27]. This reflects the median age of 9.5 years of the dogs enrolled in our study.

For an ongoing study about radiation therapy in dogs with sinonasal tumors, our group established ocular dose constraints for a 10-fraction protocol (cornea Dmax ≤ 35.4 Gy, retina Dmax ≤32.1 Gy, lacrimal glands D50% ≤ 20 Gy, eye D60% ≤ 15 Gy) based on the limited veterinary literature reports or based on human radiation oncology data [7,11,20]. Applying these constraints to the current study cohort resulted in very low rates of clinically relevant ocular toxicity: only 0.7% of eyes using VRTOG1.0 and 1.4% of eyes using VRTOG2.0. However, these dose constraints may be overly conservative, potentially limiting tumor dose escalation and compromising tumor control if strictly prioritized over optimal coverage of the target volume.

Rather than explicit radiation dose constraints, our study established TD5% and TD50% values for various acute ocular reactions in dogs. The TD5% threshold represents a relatively conservative guideline, indicating dose levels associated with a low risk of acute reactions (5% risk), whereas the TD50% threshold identifies doses associated with a significantly higher risk of developing toxicity (50% risk). In clinical practice, late toxicity is typically limited to a maximum acceptable Normal Tissue Complication Probability (NTCP) of 5% [28,29], reflecting the importance of avoiding irreversible or severe long-term complications. In contrast, higher complication probabilities may be clinically acceptable for acute toxicity, provided these effects are transient, manageable, and fully reversible without causing permanent loss of organ function or significant impairment. Currently, no universally accepted clinical threshold explicitly defines acceptable NTCP levels for acute toxicities. In clinical treatment planning, the TD5% and TD50% thresholds serve as critical reference points rather than strict dose constraints. Ideally, radiation plans should aim to keep doses below the TD5% threshold whenever feasible. However, recognizing the transient nature of acute reactions, exceeding this threshold may be clinically acceptable if required for effective tumor control – provided the potential reactions remain reversible and clinically manageable. Doses approaching or exceeding the TD50% threshold indicate a substantially increased risk and therefore require explicit clinical justification based on clinical necessity and informed owner consent.

Retinopathy is commonly considered a late radiation effect that occurs several months after RT and is irreversible [11]. However, prior clinical observations suggested that retinopathy occurs sooner in some of our patients with retinal hemorrhages appearing as a new finding as early as 3 months after RT. Indeed, minor retinal hemorrhages were already detected at the re-check examination 3 months after RT in 4 eyes of 38 dogs (7.8% of dogs, 5.2% of eyes) in this study. At this time, these minor retinopathies did not impair the dogs’ well-being, but vision impairment could become clinically evident in the future as they could be progressive in nature. Long-term follow-up with late toxicity scoring is important in those patients. Surprisingly, in our study, retinopathy grade improved in 2 of those 3 dogs at a follow-up visit.

The original modified McDonald-Shadduck scoring system was not developed to score radiation toxicity in particular [21], is very detailed and can be time-consuming. Furthermore, only a small percentage of irradiated dogs will undergo regular examinations by veterinary ophthalmologists. The VRTOG1.0 toxicity scoring system was crude and scored toxicity with only 4 grades (grade 0–3), with grade 3 representing severe ocular abnormalities including some requiring enucleation. On the other hand, the updated VRTOG2.0 toxicity scoring system includes 6 grades (grade 0–5) and is much more detailed as it lists ocular discharge, corneal, conjunctival and lacrimal abnormalities separately. Still, the VRTOG2.0 toxicity scoring system is easily usable by veterinary radiation oncologists and was *“designed for daily application”* as stated by the Veterinary Radiation Therapy Oncology Group (VRTOG), whose members (including a veterinary ophthalmologist familiar with scoring radiation oncology patients) developed the updated VRTOG2.0 version [16]. While *“not all pathologic abnormalities may be captured in this grading scheme”* according to the VRTOG, most of them should be represented, rendering this a good monitoring tool for the majority of dogs seen in daily veterinary radiation oncology practice. Indeed, clinically relevant results of the very detailed McDonald-Shadduck scoring system seem to have been accurately reflected by the VRTOG2.0 scoring system in our study. Detailed ophthalmological examinations can most likely be reserved for dogs with clinically relevant ocular abnormalities and should be used to define the optimal treatment plan.

Apart from the shortcomings already described above, the following limitations are also worth mentioning: 1) Patient numbers (n = 70) were rather small for detection of toxicity, even though patient numbers are higher than in a previous study [20] comparing lacrimal dose and occurrence of KCS (n = 15). This is even more important as only a subset of dogs developed clinically relevant ocular toxicity. 2) Not all dogs were treated with the same linear accelerator. Possible dose variations that could have appeared due to the different dose calculation algorithms between the two linear accelerators were anticipated by recalculation of all the treatment plans with the same algorithm. This rendered the dose calculations in the plans of all dogs equal. 3) The lacrimal and accessory lacrimal glands that were contoured in our study are responsible for the production of the aqueous component of the tear film. Tear film stability, however, is provided not only by this aqueous component, but also by the lipid layer produced by the Meibomian glands in the eyelid margins [26,30]. Based also on a pilot study previously performed in our clinic, we considered the presence and potential clinical impact of tear film instability after radiation therapy to be of low likelihood, and contouring of the eyelid margins unreliable, which is why these structures were not included in our contouring and dose evaluation process. 4) Radiation dose as visible in the planning system can be reported with high precision. It is important to note, however, that this does not fully represent the total dose administered to an organ at risk over 10 fractions and that this number therefore needs to be interpreted with caution. The expected small daily setup inaccuracies (positioning verification is precise, but even with advanced techniques, an uncertainty of up to 1 mm may remain) are accounted for by adding an additional margin around the tumor (planning target volume). For organs at risk, such a margin (planning organ at risk volume) can be applied as well, although rarely performed in veterinary radiation oncology. Ocular structures are very small. Inter- or intrafractional differences in patient positioning and/or positioning of the eye and eyelid closure, which vary depending on the depth of anesthesia, might have a marked influence on absorbed dose. This can be accounted for by addition of an extension margin that includes aforementioned position differences, the planning organ at risk volume. According to a previous study, the planning organ at risk volumes that would be needed to account for ocular globe, cornea, (accessory) lacrimal gland and retina are rather small [24]. The radiation dose and possible associated radiation effects are therefore most likely adequately reflected in our study. For a safer approach, a planning organ at risk volume with an additional margin added to organs at risk to account for positioning variations and physiological movement can be applied in the future as recommended in the study by Wolf et al. (Table 2). 5) All dogs received prophylactic administration of topical ocular lubricant or ointment from the onset of radiation therapy, which could mask a VRTOG2.0 grade 2 toxicity (decreased tear production or qualitative tear abnormalities treated only with lubricant). However, because repeated anesthesia itself can decrease tear production [31], the authors considered this a necessary (albeit very careful) adjunct therapy. 6) The VRTOG2.0 scoring was added retrospectively in most patients based on results of the McDonald-Shadduck scores, information from the patient chart and patient pictures. This could have introduced a bias as one score depended (partly) on another one. However, the VRTOG2.0 scoring system was published in the year 2023 [16] and was therefore not available for patients treated beforehand. 7) While most clinically relevant acute toxicities improved or resolved at the next follow-up examination, some ocular abnormalities persisted. 8) The ocular data from three radiation therapy studies about dogs with sinonasal tumors treated with a 10-fraction protocol were compiled in this acute ocular toxicity study. The recommended eye examination time points were at baseline, end of RT, and three weeks and three months following RT in all three studies and fewer time points in a subset of patients, explaining the lower number of dogs at the intervening time points. Additionally, for several dogs, owners elected not to return to our hospital for recommended follow-up examinations, resulting in loss of follow-up information. Because detailed ocular examinations are time-consuming, not all time points were recommended for dogs treated after the pilot study. Namely, the ocular examinations after 1 and 2 weeks and 1 and 2 months were omitted. In some dogs the examination at the end of radiation therapy was switched to a Schirmer tear test only, as hardly any acute ocular effects were seen at that time point due to the short radiation therapy protocol. It is therefore possible that some of the ocular toxicities, especially of lower grade, might have been missed.

The TD5% and TD50% thresholds identified in this study provide critical reference points for radiation therapy planning. Ideally, future treatment plans employing a 10-fraction protocol should aim to maintain radiation doses below the established TD5% (and TD50%) threshold to minimize toxicity risk.

A possible influence of (brachycephalic) breed or co-morbidities on the development of ocular toxicity should be evaluated in future studies. Furthermore, the late toxicity risk with adherence to the established TD5% (and TD50%) threshold values should be evaluated.

## Conclusions

In summary, the results of our assessment of ocular toxicity with three different scoring systems after irradiation with a 10-fraction protocol showed rather mild and only rarely clinically relevant ocular toxicity. Clinically relevant results of the very detailed modified McDonald-Shadduck scoring system seem to be accurately reflected by the VRTOG2.0 scoring system, which is more easily applicable by non-ophthalmologists and therefore more practically applicable for daily clinical assessments. Detailed ocular evaluations are likely to be recommended for clinically relevant VRTOG2.0 grade ≥3 toxicity to define an optimal treatment plan. Adherence to institutional ocular dose constraints significantly minimizes the risk of clinically relevant ocular toxicity.

## Supporting information

S1 Fig Modified McDonald-Shadduck ophthalmic examination protocol and grading system. (PDF)

S2 Fig Explanation of the modified McDonald-Shadduck ophthalmic examination protocol and grading system. (PDF)

S1 Table Materials used for complete ocular examinations. (DOCX)

S3 Fig Results of IOP, STT, and TBUT at various time points. The median values of intraocular pressure (IOP), Schirmer tear test (STT), and tear film break-up time (TBUT) are shown on the y-axis and the different recheck time points are shown on the x-axis. The asterisks indicate the time points with significantly lower IOP and STT values (p < 0.0001).
(TIF)
