# Supplementary material for: Evaluation of acute ocular toxicity after definitive-intent radiation therapy in canine sinonasal tumors
Source: PLoS One. 2025 Aug 11;20(8):e0329073. doi: 10.1371/journal.pone.0329073 (PMC12338778; doi:10.1371/journal.pone.0329073)
Supplement: S1 Fig — (PDF) [file pone.0329073.s001.pdf]

Ophthalmic examination protocol
nasal tumor radiation therapy study

First RT Treatment:____________________

Examination: pre-RT ☐ post-RT ☐ **Number of weeks post RT:** ____________________

Examiner: AR ☐ SP ☐ **Signature:**____________________

Date of examination:____________________

Method:

- Slitlamp Biomicroscopy (Kowa SL-17, 10x)
- Rebound Tonometry (Tonovet, three low variance measurements: record lowest measurement)
- STT
- Fluorescein

<u>0 =</u> No area of fluorescein staining.
<u>1 =</u> 1 to 25% area of fluorescein staining.
<u>2 =</u> 26 to 50% area of fluorescein staining.
<u>3 =</u> 51 to 75% area of fluorescein staining.
<u>4 =</u> 75 to 100% area of fluorescein staining.

- Rose Bengal (scoring as for fluorescein staining)
- TFBUT (directly after fluorescein application, prior to rinsing excess fluorescein out of eye: let blink to create even tear film, then open, wait and count until first discontinuities in tear film appear)
- corneal sensitivity with cotton tipped applicator (lightly touch central cornea 3x and note number of responses)
- Tropicamide dilatation
- Slitlamp Biomicroscopy of lens (Kowa SL-17, 10x)
- Indirect Ophthalmoscopy (20, 2.2D lens) post-dilatation.

Photos: exterior ocular structures ☐
fundus ☐

	**OD**	**OS**
**Tonometry:**	________**mmHg**	________**mmHg**
**STT:**	________**mm/min**	________**mm/min**
**TFBUT:**	________**sec**	________**sec**
**Fluorescein:**	0☐ 1☐ 2☐ 3☐ 4☐	0☐ 1☐ 2☐ 3☐ 4☐
**Rose Bengal:**	0☐ 1☐ 2☐ 3☐ 4☐	0☐ 1☐ 2☐ 3☐ 4☐
**Corneal sensitivity:**	________	________

Footnotes exam:

1. Corneal opacities (edema, infiltrates, deposits, fibrosis, pigmentation and neovascularization) need to be marked and drawn in the schedule below.
2. Lens: location (capsular, cortical, nuclear) and extension (punctate, incipient, incomplete, complete, resorbing) need to be marked and drawn in the schedule below.
3. Retinal detachments and areas of retinal degeneration: the location (central, mid-peripheral, peripheral, nasal, temporal, inferior, superior) needs to be marked and drawn in the schedule below.
4. Retinal tears, hemorrhages and chorioretinitis lesions: the location (central, mid-peripheral, peripheral, nasal, temporal, inferior, superior) and size (in # of optic nerve diameters (OND)) need to be marked and drawn in the schedule below.

**Right eye (OD)**	**Left eye (OS)**

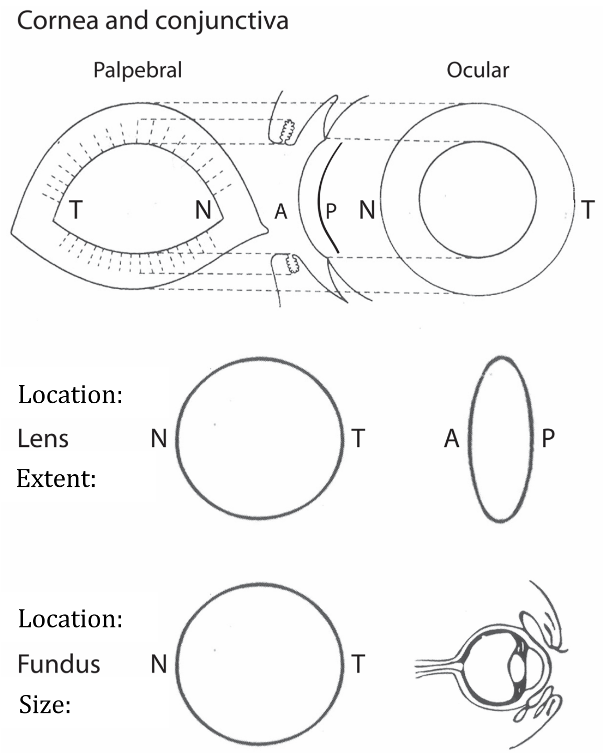

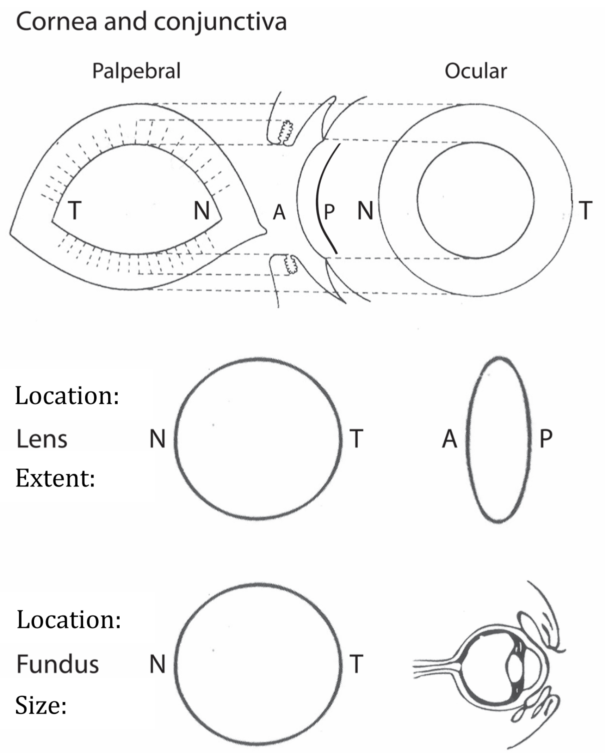

**1.** Menace response:	OD: 0☐ 1☐ 2☐	OS: 0☐ 1☐ 2☐
**2.** Dazzle reflex:	OD: 0☐ 1☐ 2☐	OS: 0☐ 1☐ 2☐
**3.** Palpebral reflex:	OD: 0☐ 1☐ 2☐	OS: 0☐ 1☐ 2☐
**4.** Pupillary light reflex direct:	OD: 0☐ 1☐ 2☐	OS: 0☐ 1☐ 2☐
**5.** Pupillary light reflex indirect:	OD: 0☐ 1☐ 2☐	OS: 0☐ 1☐ 2☐
**6.** Conjuctival congestion:	OD: 0☐ 1☐ 2☐	OS: 0☐ 1☐ 2☐
**7.** Conjuctival chemosis:	OD: 0☐ 1☐ 2☐	OS: 0☐ 1☐ 2☐
**8.** Conjuctival discharge:	OD: 0☐ 1☐ 2☐ 3☐ 4☐	OS: 0☐ 1☐ 2☐ 3☐ 4☐
**9.** Corneal opacity:	OD: 0☐ 1☐ 2☐ 3☐ 4☐	OS: 0☐ 1☐ 2☐ 3☐ 4☐
**10.** Area of corneal opacity:	OD: 0☐ 1☐ 2☐ 3☐ 4☐	OS: 0☐ 1☐ 2☐ 3☐ 4☐
**11.** Corneal pigmentation:	OD: 0☐ 1☐ 2☐ 3☐ 4☐	OS: 0☐ 1☐ 2☐ 3☐ 4☐
**12.**Corneal neovascularization:	OD: 0☐ 1☐ 2☐	OS: 0☐ 1☐ 2☐
**13.**Anterior chamber flare:	OD: 0☐ trace☐ 1☐ 2-3☐ 4☐	OS: 0☐ trace☐ 1☐ 2-3☐ 4☐
**14.** Aqueous cell:	OD: 0☐ trace☐ 1☐ 2-3☐ 4☐	OS: 0☐ trace☐ 1☐ 2-3☐ 4☐
**15.** Cell color:	OD: white☐ red☐ brown☐ mix☐	OS: white☐ red☐ brown☐ mix☐
**16.** Iris:	OD: 0☐ 1☐ 2☐ 3☐	OS: 0☐ 1☐ 2☐ 3☐
**17.** Lens:	OD: 0☐ 1☐	OS: 0☐ 1☐
**18.** Vitreal cell:	OD: 0☐ trace☐ 1☐ 2-3☐ 4☐	OS: 0☐ trace☐ 1☐ 2-3☐ 4☐
**19.** Cell color:	OD: white☐ red☐ brown☐ mix☐	OS: white☐ red☐ brown☐ mix☐
**20.** Vitreal degeneration:	OD: 0☐ 1☐ 2☐	OS: 0☐ 1☐ 2☐
**21.** Vitreal hemorrhage:	OD: 0☐ 1☐ 2☐	OS: 0☐ 1☐ 2☐
**22.** Retinal detachment:	OD: 0☐ 1☐ 2☐ 3☐ 4☐ flat☐ bullous☐ transsudate☐ exsudate☐ blood☐	
	OS : 0☐ 1☐ 2☐ 3☐ 4☐ flat☐ bullous☐ transsudate☐ exsudate☐ blood☐	
**23.** Retinal tears/holes:	OD: 0☐ 1☐	OS: 0☐ 1☐
**24.** Retinal hemorrhages:	OD: 0☐ 1☐	OS: 0☐ 1☐
**25.** Chorioretinitis lesions:	OD: 0☐ 1☐ Active☐ Inactive☐	OS: 0☐ 1☐ Active☐ Inactive☐
**26.** Retinal vessel attenuation:	OD: 0☐ 1☐ 2☐	OS: 0☐ 1☐ 2☐
**27.** Optic nerve atrophy:	OD: 0☐ 1☐	OS: 0☐ 1☐
**28.** Optic nerve cupping:	OD: 0☐ 1☐	OS: 0☐ 1☐
**29.** Retinal degeneration:	OD: 0☐ 1☐ 2☐ 3☐	OS: 0☐ 1☐ 2☐ 3☐
