# Supplementary material for: Evaluation of acute ocular toxicity after definitive-intent radiation therapy in canine sinonasal tumors
Source: PLoS One. 2025 Aug 11;20(8):e0329073. doi: 10.1371/journal.pone.0329073 (PMC12338778; doi:10.1371/journal.pone.0329073)
Supplement: S1 Table — (DOCX) [file pone.0329073.s003.docx]

**Supplementary Table 2. Materials used for complete ocular examinations.**

| **Test** | **Material** |
| --- | --- |
| Tonometry (normal range 10-25 mm/min) | Tonovet® Type TV01, Icare, Tiolat Oy, Finland |
| Schirmer tear test (normal range 10-25 mm/min) | Schirmer Tear Test; Intervet/Schering-Plough Animal Health, Roseland, NJ, USA |
| Slit-lamp biomicroscopy | Kowa SL-17 portable slit-lamp, Kowa Company Ltd, Tokyo, Japan |
| Fluorescein test | Fluo® strips, Contacare Ophthalmics and Diagnostics, Gujarat, India |
| Rose bengal staining | RoseTouch® Rose Bengal Strips, Madhu Instruments Pvt. Ltd., New Delhi, India |
| Tear film break-up time assessment | Fluo® strips, Contacare Ophthalmics and Diagnostics, Gujarat, India |
| Binocular indirect ophthalmoscopy | mPack Unplugged, Heine, Germany  using a 20 or 28 D, or a 2.2 panretinal indirect ophthalmoscopy lens (Volk® 20, 28 D, 2.2 panretinal bio lens, Ohio, USA) |
| Pupil dilation | Tropicamide 0.5 %, Théa Pharma SA, Schaffhausen, Switzerland |
| Anterior segment photography | Nikon D90 camera and Nikon AF Micro Nikkor 60 mm lens; Nikon Z6 camera and Nikon AF-S VR Micro Nikkor 105mm lens; Nikon AG, Egg, Switzerland |
| Fundus photography | Optomed Smartscope MS, Optomed Oy, Oulu, Finland 2017-2022 or Optomed Aurora Retinal Module, Optomed Oy, Oulu, Finland 2022-2023 |
