# Supplementary figures and images for: Evaluation of acute ocular toxicity after definitive-intent radiation therapy in canine sinonasal tumors
Source: PLoS One. 2025 Aug 11;20(8):e0329073. doi: 10.1371/journal.pone.0329073 (PMC12338778; doi:10.1371/journal.pone.0329073)

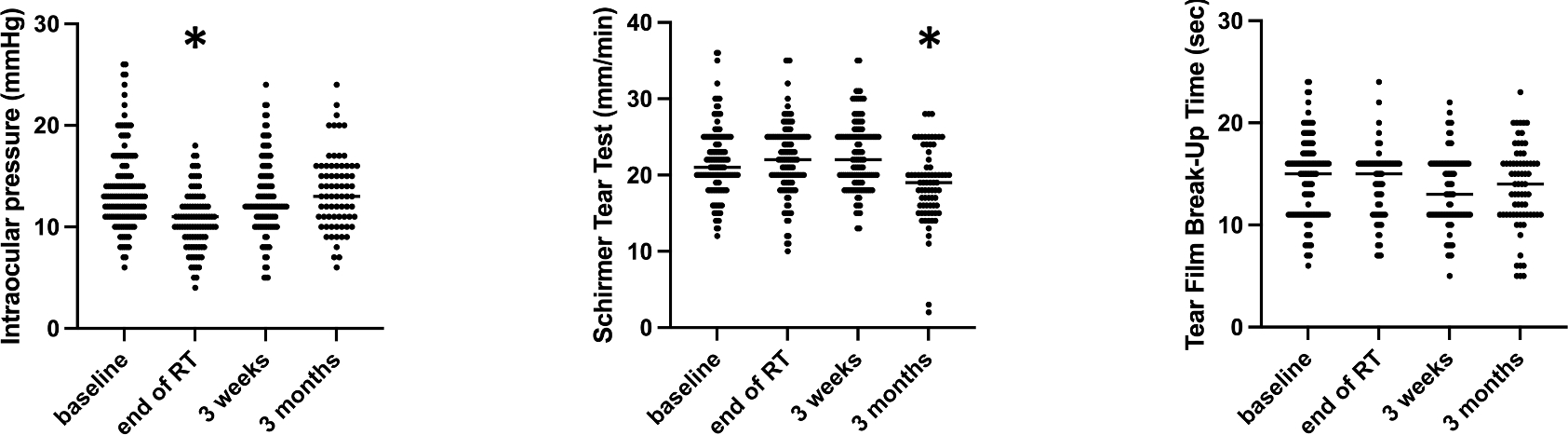

Supplement: S3 Fig — The median values of intraocular pressure (IOP), Schirmer tear test (STT), and tear film break-up time (TBUT) are shown on the y-axis and the different recheck time points are shown on the x-axis. The asterisks indicate the time points with significantly lower IOP and STT values (p < 0.0001). (TIF) [file pone.0329073.s004.tif]
